# fuseMLR: an R package for integrative prediction modeling of multi-omics data

**DOI:** 10.1186/s12859-025-06248-4

**Published:** 2025-08-26

**Authors:** Césaire J. K. Fouodo, Marina Bleskina, Silke Szymczak

**Affiliations:** Institut für Medizinische Biometrie und Statistik, Universität zu Lübeck and Universitätsklinikum Schleswig-Holstein, Campus Lübeck, Ratzeburger Allee, 23562 Lübeck, Schleswig-Holstein Germany

**Keywords:** Multi-omics, Variable selection, Integrative predictive modeling

## Abstract

****Background**:**

Recent technological advances have enabled the simultaneous collection of multi-omics data, i.e., different types or modalities of molecular data. Integrative predictive modeling of such data is particularly challenging. Ideally, data from the different modalities are measured in the same individuals, allowing for early or intermediate integrative techniques. However, they are often not applicable when patient data only partially overlap, which requires either reducing the datasets or imputing missing values. Additionally, the diversity of data modalities may necessitate specific statistical methods rather than applying the same method across all modalities. Late integration modeling approaches analyze each data modality separately to obtain modality-specific predictions. These predictions are then aggregated into a meta-model by training a machine learning (ML) model, or by computing the weighted mean of modality-specific predictions.

****Results**:**

We introduce the R package fuseMLR for late integration prediction modeling. The package is user-friendly, enables variable selection and the application of different ML algorithms for each modality, and automatically performs aggregation once modality-specific training is completed. We illustrate the package’s functionality in a small simulation study and with two publicly available multi-omics datasets from The Cancer Genome Atlas.

****Conclusion**:**

The package fuseMLR enables predictive modeling with late integration in a systematic, structured, and reproducible way.

**Supplementary Information:**

The online version contains supplementary material available at 10.1186/s12859-025-06248-4.

## Background

Precision medicine aims to improve the diagnosis, prognosis, and treatment of complex diseases by collecting and measuring several data types or modalities in addition to standard clinical information. A typical example is multi-omics data, i.e. different modalities of molecular data including genomics, epigenomics, transcriptomics, proteomics and metabolomics. Integrating such heterogeneous high-dimensional datasets when developing models to predict disease risk, disease course, or treatment response is challenging. Techniques of deep learning (DL) and large language (LL) approaches have been adapted for the integration of tabular data, e.g. by employing dimensionality reduction techniques [1]. However, tree-based methods are still the gold standard, particularly for high-dimensional data [2]. Not only are they faster and easier to use and tune, they also require fewer data to achieve high performance compared to DL approaches [3].

Among machine learning (ML) approaches, some methods work well on a specific modality but can perform poorly on others, for multiple reasons, including different scales, distributions, and correlation patterns. In addition, some of the modalities might contain redundant information or the information is irrelevant for a specific research question [4]. Furthermore, multi-omics data often exhibits a particular type of missing values, termed modality-wise missingness (or block-wise missingness), whereby one or several modalities are absent for some individuals [5].

Integrative prediction modeling approaches can be categorized according to their integration strategy [6, 7]. In early or concatenation-based integration, data from the different modalities are concatenated into a single dataset, and a prediction model is trained on the combined data. This approach has several disadvantages. First, it often does not account for the unique characteristics of individual data modalities. Second, the dimensionality increases substantially. Third, additional correlation between predictor variables can be introduced. Fourth, early integration may require additional imputation steps in case of modality-wise missingness [5]. Intermediate or transformation-based integration avoids concatenating the original data by transforming the data, e.g. into a latent space or to networks, and trains the model on the combined transformed data. Although this method can mitigate some of the challenges, it still faces limitations when data modalities are of different types or when not all modalities are available for each individual. Late or model-based integration methods first train modality-specific prediction models and thus can be based on different sets of individuals. Furthermore, different statistical or ML approaches can be applied for each modality, thereby, taking into account their specific characteristics. Predictions of the modality-specific models are then used to train meta-models using different types of aggregation methods.

Aggregation methods in late integration analysis can be classified as either simple or sophisticated. Simple methods include a linear combination of predictors, e.g., (weighted) mean or weight optimization as in the superLearner approach [8]. The linear combination of predictors as originally developed for superLearner involves training various ML models on a single dataset, assessing each method using cross-validation, and using estimations of a performance measure (e.g., the Brier score) to compute a weighted average of model-specific predictions. Motivated by the same idea, the weighted average approach can be extended to model integration for multi-omics data analysis with the difference that the weights are estimated for each data-modality. More complex aggregation methods may leverage ML techniques trained on modality-specific predictions, such as random forests (RF), least absolute shrinkage and selection operator (Lasso), or the so-called COmBined Regression Alternative (COBRA, [9]), a regression-free combination approach of model-specific predictions. The COBRA aggregation method has originally been proposed for the same setting as the superLearner method [10].

We introduce fuseMLR, an R package for performing late integration based prediction modeling of multi-omics and other multi-modal data. This package provides a framework to apply the different steps, including modality-specific variable selection, modality-specific model training, meta-model training, and making predictions in a valid, structured, flexible, and reproducible way. Various R packages and functions for variable selection, modality-specific model training and meta-model training can be used; moreover, users can easily incorporate other methods as needed. We demonstrate the package’s functionality using both simulation and case studies.

## Implementation

**Fig. 1:**
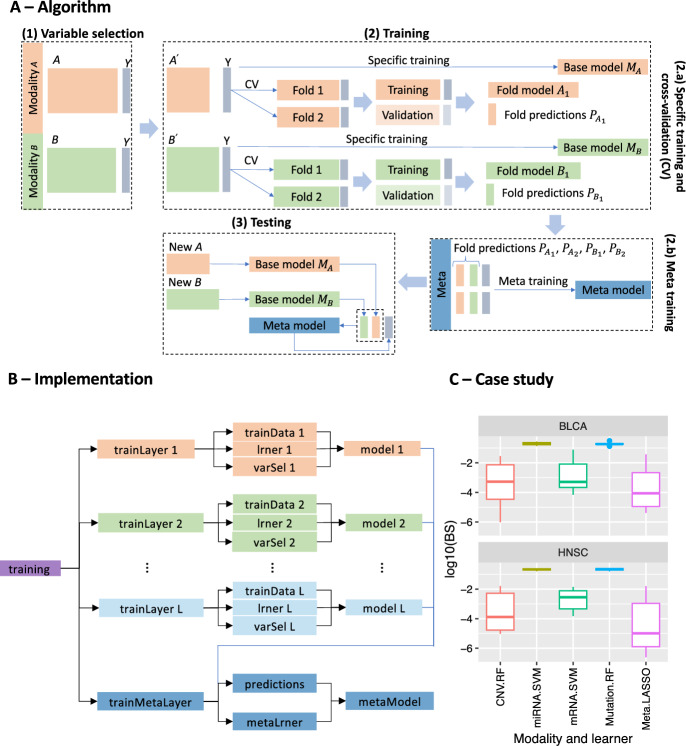
Overview of the R package fuseMLR. **A** Algorithm: Training and testing the late integration prediction model. **B** Implementation: Package components and connections. **C** Case study: Prediction performance for the modality-specific models and the meta-models trained and tested on TCGA datasets for bladder (BLCA) and head and neck squamous cell carcinoma (HNSC) cancer. Modality names followed by learner names are showed on the *x*-axis. The *y*-axis shows the log-transformed Brier scores

### Algorithm

An overview of the algorithm implemented in the package is given in Fig. 1A. For illustration, we consider a training dataset with two data modalities *A* and *B*, and the outcome variable *Y*. The training process optionally begins with variable selection, resulting in the reduced training data $A^{\prime }$ and $B^{\prime }$. For each, a modality-specific base model is trained and stored (base model $M_A$ and base model $M_B$). To avoid overfitting, this model can not be used to generate predictions as input to train the meta-model. Thus, a *k*-fold cross-validation (CV) ($k = 2$ only for illustration) is applied to split the data into training and validation folds. The fold models $A_1$ and $B_1$ developed on the training folds are used to generate predictions for the validation folds ($A_1$ and $B_1$). The meta-model is then trained based on the concatenated CV-based predictions using an aggregation method. When predicting the outcome of an independent observation, the base models $M_A$ and $M_B$ are applied to get modality-specific predictions, which are then used as input for the meta-model.

Our algorithm shares similarities with the superLearner [8] and ensemble learning stacking (ELS) algorithm [11], as all aim to train multiple learners and aggregate their predictions into a final output. However, important differences exist. First, unlike ELS and superLearner, which operate on a single dataset, our method can handle multiple data-modalities. Second, our algorithm incorporates variable selection, which superLearner and ELS do not. Third, although all methods utilize cross-validation (CV) to generate model predictions and mitigate overfitting, they differ in how they train the final meta-model. Whereas our method uses models trained on the full data modalities to generate predictions for new data, analogous to ELS for one dataset, the ELS algorithm relies on CV-based models, producing as many predictions as there are CV folds, which are then aggregated.

### Main classes

The implementation of the algorithm is based on R6 classes. The two main classes are Training and Testing , which contain the relevant information for training and testing the late integration prediction model. Figure 1B provides an overview of a Training instance (denoted as training), and its associated components, each one being an instance of a class. A training contains several training layers (*L* instances in this example) and a training meta-layer, implemented by TrainLayer and TrainMetaLayer classes, respectively. Each trainLayer *l* refers to one data modality and encapsulates the modality-specific training data trainData *l* (from the TrainData class), an R function implementing a statistical or ML approach lrner *l* (from the Lrner class) and a variable selection algorithm varSel *l* (from the VarSel class). The training meta-layer is a special layer. It is designed to contain the input for the meta-model, the modality-specific predictions (predictions from the Predictions class) and the aggregation method (metaLrner from the MetaLrner class). In addition, each training layer and the training meta-layer instance includes a modality-specific model (model *l*) and a meta-model (metaModel), implemented by the classes Model and MetaModel respectively. The second main class Testing is used to make predictions on independent testing data. It contains only testing layers and a testing meta-layer without learners or variable selection methods.

### Package functionalities

In addition to the R6 classes, the R package fuseMLR contains several standard R functions to create a training instance (function createTraining), to add a trainLayer (function createTrainLayer) or a meta-layer (function createTrainMetaLayer), to perform variable selection (function variableSelection), to train a late integration prediction model (function fusemlr), and to generate predictions for independent observations (function predict).

The package can be used with any variable selection function or (meta)learner using different approaches. If the minimum requirements for arguments and output format are not met, the user may choose to map different arguments or provide wrapper functions (for details see the vignette available on CRAN https://cran.r-project.org/web/packages/fuseMLR/vignettes/fuseMLR.html).

We incorporate the Boruta variable selection approach [12], which can be used without additional mapping or wrapping steps. Degenhardt et al. [13] have recommended this approach as a well-performing RF-based variable selection method, particularly in high-dimensional settings. The main idea of this iterative algorithm is to estimate the importance of the original predictor variables and to compare them to the importance estimated of the so-called shadow variables, obtained by permuting the original variables. At each iteration, for each independent variable, it is counted if the estimated importance is larger than the maximum among the importance estimated from all shadow variables. Subsequently, a binomial test with a given significance level is used for each predictor variable to test whether this count is larger than expected by chance. Beyond the Boruta variable selection method, fuseMLR’s users can wrap or incorporate any other variable selection methods as explained in the package vignette.

ML methods can be utilized similarly, with the option of selecting different approaches for each modality and the meta-model. We provide the random forest (RF) implementation in the R package ranger [14] as an easy-to-use example. Currently implemented aggregation methods include simple approaches such as selecting the best performing model or weighted and unweighted averaging, standard ML approaches such as RF, and the COBRA [9] method. The implemented aggregation methods are designed for both complete and missing modality-specific predictions. They also share the advantage of being able to deal well with highly correlated predictor variables, which, in this case, are the predictions of the modality-specific models.

The training of the simple aggregation methods is based on prediction performance. We allow the user to choose which performance measure to use, with the Brier score being the default. For the weighted average, the weights are inversely proportional to the Brier score, so the larger weight is assigned to the modality-specific model with the lowest Brier score. For observations with missing modalities, we weight and average the predicted values for the available predicted modality-specific predictions. When aggregating modality-specific predictions using the best learner, we handle missing values by recursively choosing the value predicted by the model ranked as the next-best model if the value predicted by the best model is missing.

The RF algorithm can internally deal with missing values if a current version of ranger ($\ge$ 0.17.0) is used. The main idea is to ignore missing values when finding the best split point of the current split variable. For the selected threshold, it is determined if all observations with missing values should be assigned to the left or right child node, and the resulting node direction is saved. For prediction, if a missing value occurs at a node without a default direction, the corresponding individual is assigned to the left node.

The implementation of the fuseMLR method in fuseMLR allows training the meta-model on the meta-data with missing values without additional imputation steps. It is a regression-free prediction method that uses a collection of basic estimators to indicate the proximity between the training data and a test observation. For a given test observation, the COBRA algorithm estimates the distances between the test observation and the training data based on complete information, i.e., by ignoring missing values. Subsequently, the prediction of the test observation is made by choosing the majority class or estimating the frequency of the majority class from its neighborhood.

### Illustrative example

A complete workflow for the use of fuseMLR includes creating a training and a testing object. For illustration, we used the simulated datasets multi_omics, published with the package. Data have been simulated using the R package InterSIM, designed for simulating multi-omics data [15]. The multi_omics object includes a list of training omics-datasets and another list with testing data saved as data.frame. The following code is a skeletal description of how to use the fuseMLR package to develop a late integration prediction model. A complete version is provided in our online vignette (https://cran.r-project.org/web/packages/fuseMLR/vignettes/fuseMLR.html).

We start by loading the package and creating an empty training object with the createTraining function. Requirements are the training object’s ID, the column ID’s name in each data-modality, and the vector of observed values for the dependent variable.
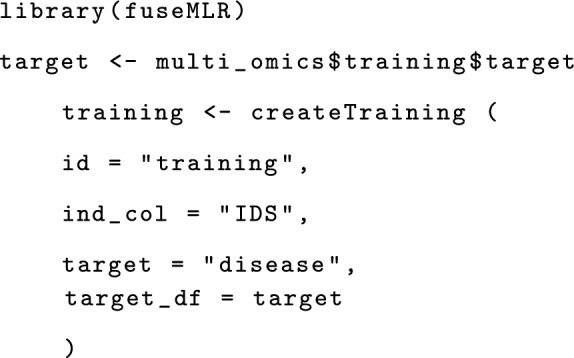


We can set up TrainLayers to the training object using the createTrainLayer function. This includes adding a data modality, a variable selection function, and a learner. For this example, a gene expression data modality is considered. The variable selection function is Boruta and the learner is ranger. The parameters of the variable selection function and the learner are passed as list objects. The optional parameter na_action specifies how to handle missing values. For this example, we set its value to na.keep, meaning that the learner ranger will internally handle missing values. We add only one TrainLayer, but more TrainLayers can be added analogously.
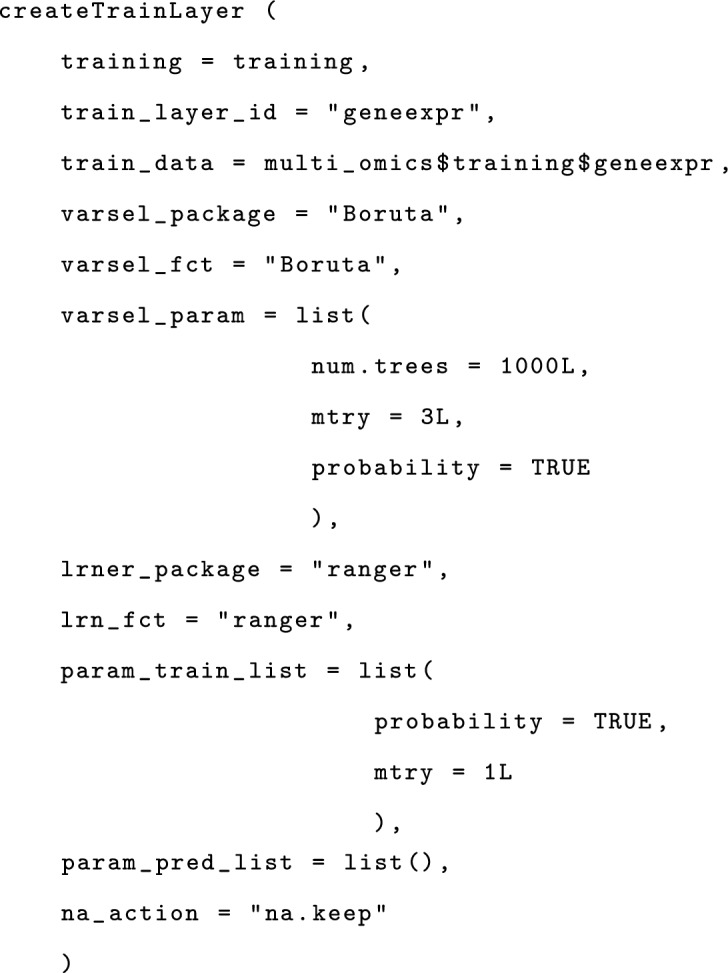


The function createTrainMetaLayer for creating a TrainMetaLayer, takes as parameters a training object, an ID, eventually the package implementing the learner (NULL for the current example since the learner weightedMeanLearner we use is implemented by fuseMLR), the training and predicting parameters for the meta-learner, and the parameter na_action specifying how to handle missing values which is again set to na.keep, since our implemented meta-learner (weightedMeanLearner) can handle missing values.
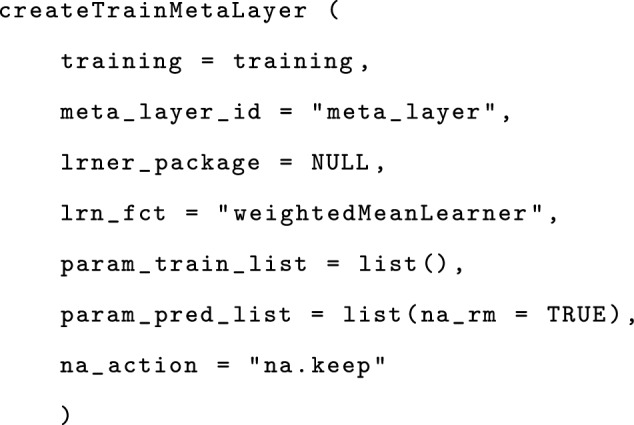


Once training has been completely set up, variable selection can be performed, followed by training using the function fusemlr. The parameter use_var_sel specifies that variable selection is required before training.
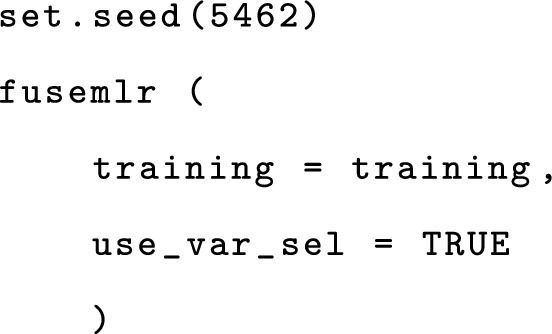


Functions extractModel() and extractData can be used to extract trained models, data modalities, and meta-data.

A testing object is required for prediction of new data modalities. It is created using the createTesting function, analogous to createTraining.
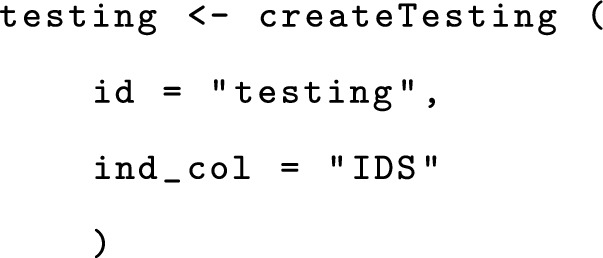


The function createTestLayer to create TestLayers requires an ID (matching the corresponding TrainLayer in the training), and the testing data modality. The following example adds the gene expression modality to the testing object. Other data modalities can be added analogously.
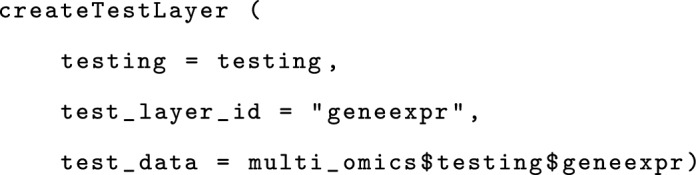


Finally, we can predict the outcome for the testing object as follows:
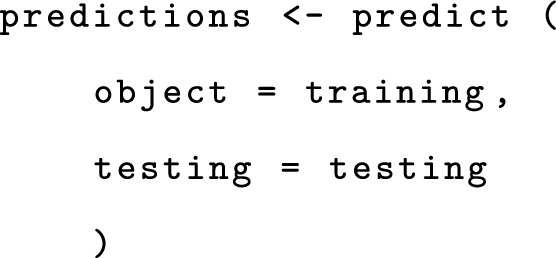


## Results

### Simulation study

We conducted a simulation study to evaluate the effectiveness of fuseMLR as a multi-omics prediction modeling approach for late integration.

**Fig. 2:**
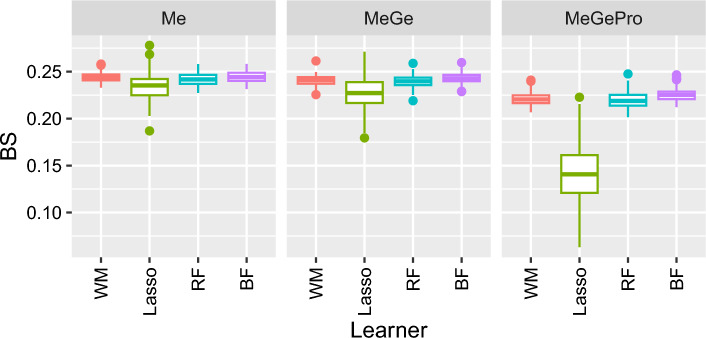
Estimated Brier score (BS) is shown for the three scenarios with effect in methylation only (Me), effect in methylation and gene expression (MeGe), and effect in methylation, gene expression and protein abundance (MeGePro) modalities. On the *x*-axis meta-learners are weighted mean (WM) and Lasso for late integration with fuseMLR, and random forests (RF) and BlockForest for the early integration approaches

We used the R package InterSIM to simulate data-modalities from three multivariate normal distributions [15], and with empirical means and variance-covariance matrices estimated based on the ovarian cancer study from The Cancer Genome Atlas (TCGA) [16]. The simulated datasets include methylation values for 367 CpGs, expression values for 131 genes, and abundance of 160 proteins. We simulated 300 individuals with complete data for each modality, and used 200 for model training and 100 for testing. We used a balanced ratio of cases and controls by randomly selecting 20% of the predictor variables in each data modality to have an effect. An effect size of 0.1 was simulated for each relevant predictor variable. We distinguished three different scenarios: one in which only the methylation data-modality has effects (Me), one in which both methylation and gene expression have effects (MeGe), and a third in which all three modalities have effects (MeGePro). For comparison, we included early integration methods, the standard random forest (RF) and block forest (BF) [17]. The primary difference between these methods lies in how they handle the modality structure (also referred to as blocks in BF) during the selection of split variables. The standard RF ignores the block structure of the concatenated dataset, which can bias the selection toward variables of larger modalities. In contrast, BF explicitly accounts for the modality structure by first selecting a block uniformly at random and then choosing a split variable from within that block, thereby ensuring that all modalities contribute equally, regardless of their dimensionality. We conducted 100 runs for each scenario. For each run, we performed late integration prediction modeling using fuseMLR, and early integration prediction modeling using a standard RF and BF with 5000 trees. For fuseMLR, we fitted an RF model for each data-modality. As meta-models, we considered a least absolute shrinkage and selection operator (Lasso), as implemented in the glmnet package [18] and a weighted average model. Subsequently, we predicted the probability that each individual in the test set is a case. We used the Brier score (BS) [19], the F-score, and the Area Under the Curve (AUC) to assess the performance of the models in each scenario. Good model performance is given by low BS, but high F-score and AUC. Figure 2 shows the estimated BS of the simulation study. The estimated F-score and AUC are presented in the supplementary file.

The fuseMLR’s meta-model, using Lasso as the meta-learner, outperforms all other approaches with the lowest BS, and across all scenarios. The most considerable difference is observed for the MeGePro scenario, for which all three modalities (methylation, gene expression, and protein abundance) contain effects. As more modalities contribute to prediction, the difference between lasso-based late integration and the other approaches increases. The three other approaches have similar performance, however, for all scenarios, the BF performance is the lowest. The model ranking observed for BS as a performance measure was similar to that observed for F-score and AUC.

### Case study

We used two multi-omics datasets from TCGA [16] to further illustrate the functionality of the fuseMLR package.

We used the available version of the data processed by Herrmann et al. [20]. The first dataset focuses on bladder cancer (BLCA), with information from 382 patients. The second dataset includes 443 patients with head and neck squamous cell carcinoma (HNSC), a type of cancer that affects the oral cavity. For both datasets, we used four omics modalities: CNV (copy number variations), miRNA, mRNA, and mutations. Apart from the miRNA modality with less than 1000 variables in both studies, all other modalities contain at least 15,000 variables, with the CNV data having more than 50,000 variables. Data on each modality was available for all individuals. Similarly to previous studies [21, 22], we considered sex as the outcome variable. Although it is not a clinically meaningful outcome, it is expected that the biological signal contained in some of the omics modaities is strong, so that the outcome can be predicted with high accuracy [22]. This makes it a suitable outcome variable for illustration purposes. For CNV and mutations with ordinal and binary predictor variables, we employed the RF algorithm as the base learner using theranger implementation [14] with 25,000 decision trees. For miRNA and mRNA we utilized support vector machines with a radial basis kernel, implemented in the R package e1071 [23]. As meta-learner, we apply the Lasso from the glmnet package [18]. We evaluated the prediction performance of both modality-specific models and the meta-learner using a 10-fold CV and the BS [19], with lower values of BS indicating better performance. In Fig. 1 C the logarithmic transformed BS ($\log _{10} (BS)$) is shown for better visualization, since the BS is large for some modalities (miRNA and mutation), but very small for the other modalities (CNV, mRNA) and the meta-learner. The different performances of the modality-specific models can be partly explained by the number of predictor variables located on the X and Y chromosomes. In contrast to more than 2800 CNV positions (= 4.9%), 740 (= 3.5%) protein-coding genes, 720 (= 4.0%) mutations, only less than 65 (= 8.0%) of miRNAs were from these chromosomes. Of note, the meta-learner outperforms the modality-specific learners in each of the two studies. The CV was run on a 64-bit Linux platform with two 16-core Intel Xeon E5-2698 v3 CPUs. The average runtime per CV fold was 8.78 minutes for BLCA and 9.6 minutes for HNSC. We did not report the F-score and the AUC in this case because their estimated values were equal to one for all learners.

## Discussion

The fuseMLR workflow includes variable selection, modality-specific model training, the training of a meta-model and prediction. As variable selection keeps the relevant variables for prediction, we strongly recommend this step for high-dimensional data modalities. Removing noise variables from the modality-specific data can improve the prediction performance of the modality-specific model, minimize differences in performance of modality-specific models, and reduce the impact of model choice on final prediction performance. We would also like to stress that performing variable selection separately for each modality might have the disadvantage that possible interaction effects between data modalities can be lost.

Choosing appropriate modality-specific prediction methods can be challenging. Simple regression methods may be recommended for interpretability purposes for some specific types of data modalities, such as clinical information. More sophisticated ML methods would be preferred for high-dimensional data modalities. For example, because of the expectation of highly correlated, skewed distributions of metabolites, we recommend using RF as a modality-specific method. The RF method is also suitable for CNV data modalities, as such data can be treated as ordinal or quantitative measurements, and RF can deal with both types. Concerning gene expression data, strictly known as quantitative, a wider range of ML methods can be applied as modality-specific learning methods. Given the expected marginal distribution of gene expression data, we recommend scaling observations before applying SVM. In contrast, random forests typically do not require this step, as they are robust to distributional skewness.

A lack of understanding of the specific characteristics of each data modality may hinder the selection of ML algorithms. For a general recommendation, we assume that the prediction performance of the meta-learner depends on modality-specific predictions; good modality-specific predictions are crucial for good final predictions. Therefore, modality-specific predictions obtained using the internal cross-validation implemented in fuseMLR are useful to assess modality-specific models without additional programming steps. The user can utilize these predictions to evaluate the performance of modality-specific models. A more complete framework might include an evaluation of the meta-learner’s predictions and then re-evaluate the modality-specific predictions. That is, the choice of the modality-specific learners would depend on the final predictions, not only the modality-specific predictions. Although such an approach would involve assessing the modality-specific models and the meta-learner candidates, it can result in evaluating numerous learner combinations and thereby increase computational time. Selecting the modality-specific models, followed by choosing an appropriate meta-learner, is a practical and effective approach.

Selecting a meta-learner can appear more challenging than selecting the modality-specific models. For this step in the workflow, we recommend an additional cross-validation to estimate the performance of the candidate meta-learners. However, since the initial cross-validation is already used to generate modality-specific predictions, this results in a nested cross-validation setup. Consequently, users may face limitations due to the sample size. To address this, we plan to conduct more comprehensive simulation studies to evaluate different meta-learner approaches across a wider range of settings, so that more specific recommendations can be provided.

In the case study, we considered some modality-specific learners and the Lasso prediction method as a meta-learner. As the choice of the modality-specific learner was based on the data types, the choice of Lasso as meta-learner was mainly supported by the fact that this method is designed to work with highly correlated independent variables. Based on this motivation, other prediction methods, such as RF and COBRA, might also be used as meta-learners. We recommend evaluating multiple candidate methods through internal comparison to determine the best-performing meta-learner among the learner candidates. A large sample size will be necessary for a reliable performance comparison.

Although the idea behind our proposed package shares similarities with previously introduced approaches such as superLearner [8] and the ELS algorithm [11], a direct comparison presents notable limitations. Specifically, since superLearner and ELS are designed to operate on a single dataset with multiple learners, comparing them with fuseMLR would require concatenating all data modalities into one unified input. This would undermine the core concept of late integration, which fuseMLR is explicitly designed to support. However, keeping each data modality separate prevents using superLearner or ELS, as they cannot handle multiple input sources independently.

Our work includes a comparison of the proposed late integration prediction modeling method with early integration approaches, showing a preference for the former type of approach. A broader comparison, which is beyond the scope of this manuscript, should also include intermediate integration approaches. The results of such a study would allow for recommendations regarding which context to use which integration technique, or to understand when early or intermediate integrations are advantageous for improving prediction performance. A similar question was investigated in the study by Li et al. [24] using 14 TCGA cancer datasets. They analyzed whether combining multiple omics data improves prediction performance in a survival context and concluded that adding more data modalities may reduce the prediction performance. The late integration approach with the best modality-specific learner as a meta-learner would be the recommended strategy for such a situation. This supports the choice of the appropriate meta-learner as a crucial step in the late integration modeling.

We developed a new R package for multi-omics data integration and predictive modeling, with a strong focus on ML approaches. While ML remains a core strategy, there is growing interest in adapting DL and LL models (LLM) to enhance biomedical research. For instance, [25] and [26] recently introduced DL-based methods—DeepKEGG and MULGONET—for predicting cancer recurrence. Additionally, [27] conducted a comprehensive review demonstrating a significant rise in the use of LLMs in biomedical applications. We conceived a flexible and modular implementation of our package to accommodate pre-trained models tailored to the specific modality in future versions.

## Conclusion

We present the R package fuseMLR as a late integration prediction modeling framework. Its usage is not restricted to multi-omics data or a particular combination of omics data, but can also be used with other data modalities as long as they can be stored in tabular form. The package is flexible regarding optional variable selection, algorithms for training modality-specific prediction models and aggregation methods to combine their predictions into a meta-model. Additionally, different methods can be compared by reusing already created components, without changing the formats. The implementation based on R6 classes supports statisticians, bioinformaticians, and data scientists to perform integrative prediction modeling in a systematic and reproducible way. The current version only handles classification and regression problems, but we plan to extend it to survival analysis as well.

## Supplementary Information

Supplementary Material 1.

## Data Availability

The multi-omics datasets from The Cancer Genome Atlas (TCGA) are publicly available. For the use case preprocessed data sets (IDS: BLCA, HNSC) were downloaded from OpenML (https://openml.org). The code to reproduce the results of the case study is provided on GitHub (https://github.com/imbs-hl/fuseMLR-paper). Project name: fuseMLR Project home page: CRAN (https://cran.r-project.org/web/packages/fuseMLR), GitHub (https://github.com/imbs-hl/fuseMLR/tree/master) Operating system: Platform independent Programming language: R Other requirements: R ($\ge$ 3.6.0) License: GPL-3 Any restrictions to use by non-academics: none.

## Abbreviations

ML: Machine learning
RF: Random forests
Me: Methylation
MeGe: Methylation and gene expression
MeGePro: Methylation, gene expression, and protein abundance
BF: Blockforest
COBRA: COmBined regression alternative
CV: Cross-validation
ELS: Ensemble learning stacking
TCGA: The cancer genome atlas
BLCA: Blader cancer
HNSC: Head and neck squamous cell carcinoma
CNV: Copy number variation
LASSO: Least absolute shrinkage and selection operator
BS: Brier score
AUC: Area under the curve
