# Supplementary material for: fuseMLR: an R package for integrative prediction modeling of multi-omics data
Source: BMC Bioinformatics. 2025 Aug 26;26:221. doi: 10.1186/s12859-025-06248-4 (PMC12382258; doi:10.1186/s12859-025-06248-4)
Supplement: Supplementary file 1 — Supplementary Material 1. [file 12859_2025_6248_MOESM1_ESM.pdf]

Estimated Brier score and AUC are presented for the simulation study

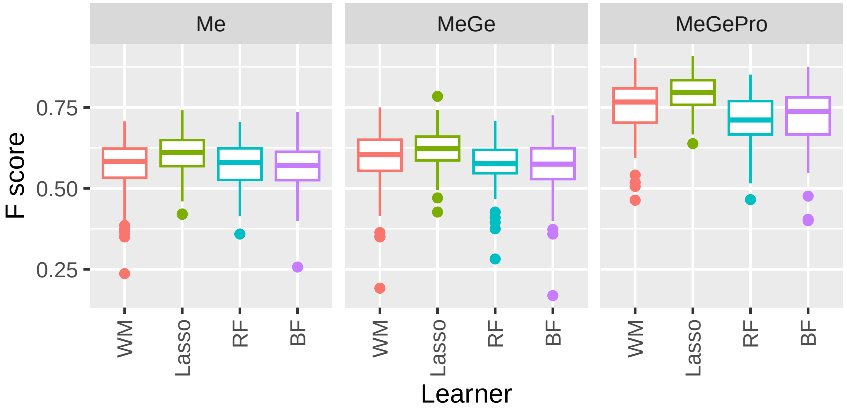

Supplementary figure 1 Estimated F-score is shown for the three scenarios with effect in methylation only (Me), effect in methylation and gene expression (MeGe), and effect and methylation, gene expression and protein abundance (MeGePro) modalities. On the x-axis meta-learners are weighted mean (WM) and Lasso for late integration with `fuseMLR`, and random forests (RF) and BlockForest for the early integration approaches.

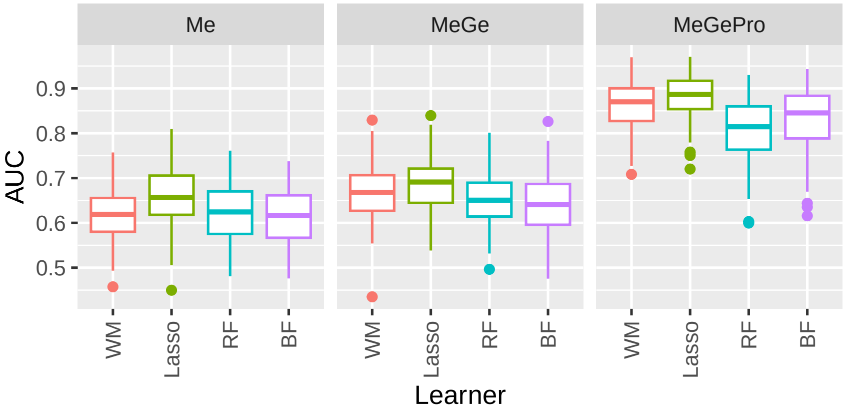

Supplementary figure 2 Estimated AUC is shown for the three scenarios with effect in methylation only (Me), effect in methylation and gene expression (MeGe), and effect and methylation, gene expression and protein abundance (MeGePro) modalities. On the x-axis meta-learners are weighted mean (WM) and Lasso for late integration with `fuseMLR`, and random forests (RF) and BlockForest for the early integration approaches.
